# Improved clinical outcome measures of knee pain and function with concurrent resolution of subchondral Bone Marrow Edema Lesion and joint effusion in an osteoarthritic patient following Pentosan Polysulphate Sodium treatment: a case report

**DOI:** 10.1186/s12891-017-1754-3

**Published:** 2017-09-12

**Authors:** Matthew J. Sampson, Margie Kabbani, Ravi Krishnan, Michael Nganga, Annika Theodoulou, Jeganath Krishnan

**Affiliations:** 1 Benson Radiology, 120 Greenhill Road, Unley, South Australia 5061 Australia; 2 The International Musculoskeletal Research Institute Inc, 13 Laffers Road, Belair, South Australia 5052 Australia; 3 Paradigm BioPharmaceuticals Ltd, Level 2, 517 Flinders Lane, Melbourne, VIC 3000 Australia; 4 College of Medicine and Public Health, Flinders University, Sturt Road, Bedford Park, South Australia 5042 Australia

**Keywords:** Bone marrow edema lesion, MRI, Knee osteoarthritis, Joint effusion; Pentosan polysulphate sodium

## Abstract

**Background:**

At present, there are no registered products for the treatment of subchondral Bone Marrow Edema Lesion (BML) and associated knee pain. Patients who do not respond to current anti-inflammatory therapies are left with limited treatment options, and may resort to operative management with Total Knee Arthroplasty (TKA). We report the use of Pentosan Polysulphate Sodium (PPS) for the treatment of BMLs of the knee.

**Case presentation:**

We report the case of a 70-year-old female with knee osteoarthritis presenting with a high level of knee pain, scoring 8 on the Numerical Rating Scale (NRS), and functional limitation demonstrating a poor Lysholm Knee Score of 37. MRI scans of the knee revealed subchondral BML in the medial femoral condyle and medial tibial plateau. The patient was administered a course of Pentosan Polysulphate Sodium (PPS) intramuscularly twice weekly, for 3 weeks. MRI scans 2 weeks post-treatment showed complete resolution of the bone marrow edema at the medial femoral condyle and medial tibial plateau with concomitant recovery from pain (NRS pain score of 0), and a 43% improvement of the Lysholm Knee Score. In addition, marked reduction in joint effusion was also demonstrated in the MRI scan post PPS therapy.

**Conclusion:**

The MRI interpretations demonstrate improved clinical outcome measures ensuing therapeutic intervention with PPS, and warranting further investigation into the efficacy of PPS in the treatment of BML associated pain and dysfunction in the osteoarthritic population via randomized controlled trial, or equivalent rigorous methodological technique.

## Background

Bone Marrow Edema Lesions (BMLs) are changes that occur in the subchondral bone and are detected by Magnetic Resonance Imaging (MRI), depicting the severity of symptoms including pain [1–3] and cartilage degeneration [4–7] in osteoarthritic patients. BMLs are generally evaluated using fat-suppressed proton density or T2-weighted sequences. In fat suppressed T2-weighted and fat suppressed proton density weighted sequences, BMLs appear as hyper-intense areas in subchondral bone and in T1-weighted sequences they appear as hypo-intense signals [8]. The MRI signals related to the BML are thought to arise from an increase in concentration of blood and interstitial fluids (including infiltrating macrophages) in areas of trabecular microfractures and collapse within the bone marrow [8]. Improved spatial resolution and multiplanar reconstructions provide a potential role of 3D fast spin echo sequences, particularly for imaging of cartilage [9].

Increasing evidence supports the notion that BMLs play an important role in the pathogenesis of knee osteoarthritis, particularly in established disease where BMLs are associated with knee pain [2], radiological progression of knee osteoarthritis [10] and cartilage loss based on MRI [5, 11]. In regards to progressive osteoarthritis, data have suggested that BMLs are more likely to persist and enlarge in size with an associated increase in cartilage loss [5]. Furthermore, the severity of BMLs has shown to correlate with the increasing risk of knee arthroplasty [12]. The strong association of BMLs with pain and loss of cartilage has heightened pharmaceutical interest to target this structural lesion for monitoring progression of knee osteoarthritis and therapeutic effects [13–15].

Pentosan Polysulphate Sodium (PPS) is a semi-synthetic drug manufactured from beech-wood hemicellulose by sulphate esterification of the xylopyranose hydroxyl groups [16]. PPS has been used in the treatment of horses with osteoarthritis, and demonstrated beneficial effects on the cartilage fibrillation and synovial fluid concentrations [17, 18]. In addition, further research into the anti-inflammatory properties of PPS have concluded (i) inhibition of the cartilage degrading enzymes which are upregulated post-acute injury [19]; (ii) inhibition of the nuclear translocation of NF-kappaB and the regulation of transcription of the pro-inflammatory cytokines TNF-alpha and interleukin IL-1 beta [20]; (iii) antithrombotic and antilipidemic effects which may assist with improved microvascular circulation in the subchondral bone [21] and considered to be mechanistically relevant in resolving BML. Furthermore, the safety profile of PPS has been further validated in two clinical trials in osteoarthritis patients [16, 22].

In this report we have observed the anti-inflammatory, disease modifying properties of PPS that potentially resulted in the rapid reduction of BML and joint effusion in an osteoarthritic patient.

## Case presentation

**Fig. 1:**
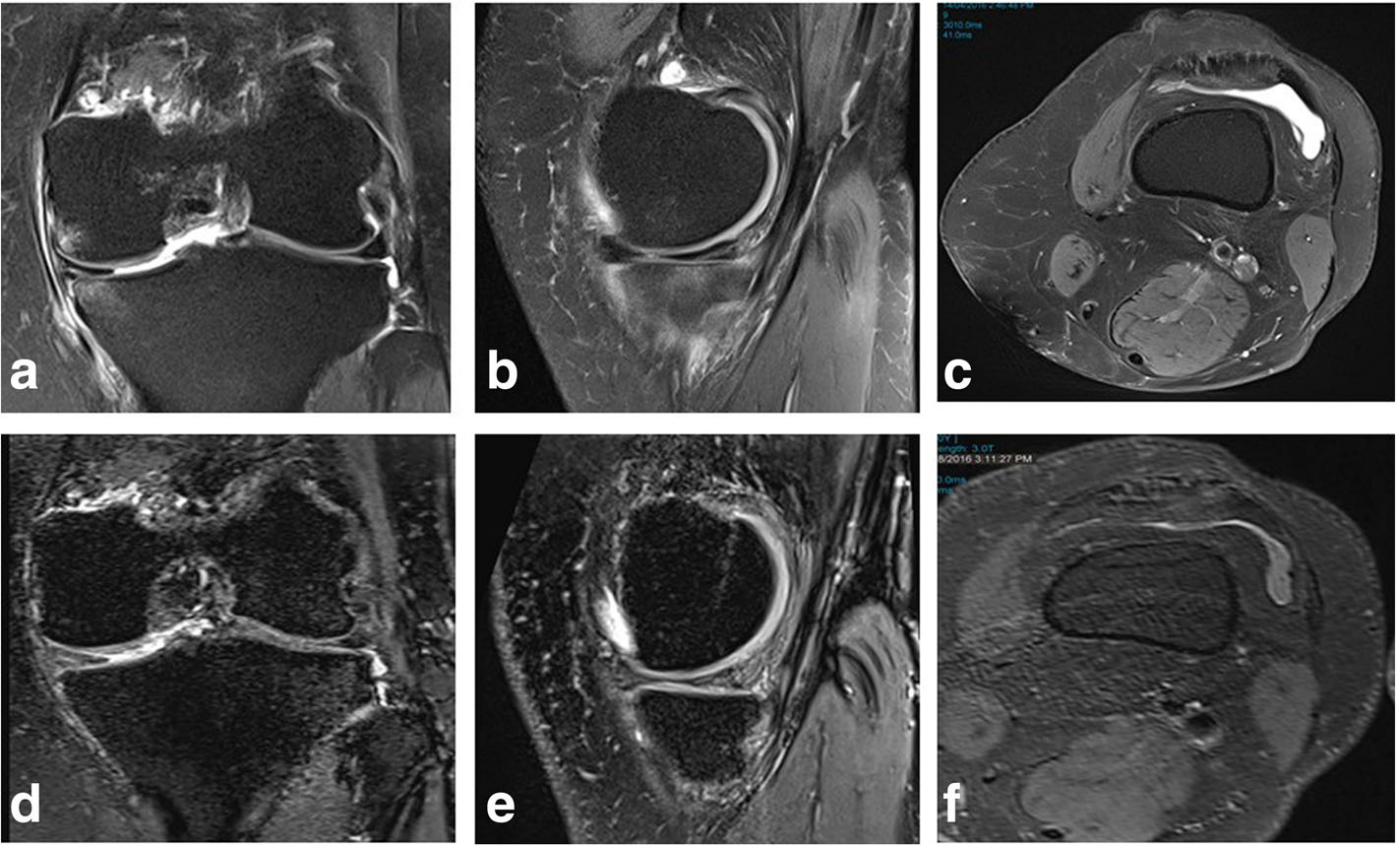
Pre-treatment 3T proton density fat saturated images in coronal (**a**) and sagittal (**b**) planes demonstrating medial compartment bone edema, more pronounced on the tibial side. Axial (**c**) proton density fat saturated imaging at the level of suprapatellar pouch demonstrating a knee effusion. Corresponding post-treatment 3T proton density fat saturated images in coronal (**d**) and sagittal (**e**) planes demonstrating resolution of medial compartment bone edema. Axial (**f**) proton density fat saturated imaging at the level of suprapatellar pouch demonstrating reduction in size of knee effusion

A 70-year-old female with a history of osteoarthritis and arthroscopic partial medial meniscectomy presented with pain of the left knee and was on a waiting list for Total Knee Arthroplasty (TKA). The patient had failed to respond to intra-articular cortisol administration at the time of arthroscopy. The MRI scan of the knee, using a 3-Tesla proton density turbo spin echo fat saturated acquisition with TR 3000 ms TE 30 ms, demonstrated subchondral BML associated with focal full thickness chondral defects at the medial aspects of the weight bearing medial femoral condyle and medial tibial plateau (Fig. 1). The BML in the medial femoral condyle measured 11 × 7 × 12 mm (CC x transverse x AP) and the medial tibial plateau BML measured 8 × 8 × 8 mm (CC x transverse x AP). In addition, the axial proton density fat saturated imaging at the level of suprapatellar pouch revealed a knee effusion and a recurrent tear of the medial meniscus (body and posterior horn). Following patient consent, the pain assessment score was determined by the Numerical Rating Scale (NRS), an 11-point scale with endpoints of ‘a state of no pain (“0”)’ and ‘the worst pain imaginable (“10”)’ [23]. Functional capacity was assessed using the Lysholm knee score [24]. At pre-treatment, the NRS pain score was severe with a score of 8 out of 10 (Scale range: 0 to 10) and the Lysholm Knee Score was 37 out of 100, reflecting poor knee function indicated by problems in stair climbing and limping.

As PPS is a weak anticoagulant with 1/15 the activity of heparin, the patient was monitored for safety by assessments of complete blood count, Activated Partial Thromboplastin Time (APTT), prothrombin time, liver function tests, renal function tests and serum calcium prior to commencement of treatment and periodically during treatment with PPS. The clinical status of the patient was checked regularly throughout the course of the treatment and follow up period.

After evaluation, the patient was considered suitable for PPS treatment. Since the injectable form of PPS is not a registered product in Australia for the treatment of BMLs, approval to use PPS as treatment for the patient was acquired from the Department of Health - Therapeutic Goods Administration, Australian Government under the Special Access Scheme. The patient was administered 2 mg/kg of PPS twice weekly with a minimum of 3 and a maximum of 4 days between dosages. Six intramuscular injections were administered into the gluteus maximus muscle over a 3-week period (2 injections per week). During the course of PPS treatment the patient abstained from NSAIDs and did not receive any additional therapy. Three follow-up appointments were made at 10, 24 and 38 days post-completion of injection regimen. Five weeks after the first PPS injection, MRI scans were performed using a 3-Tesla proton density fat saturated SPACE acquisition with TR 1200 ms TE 28 ms which demonstrated complete resolution of the BMLs at the medial femoral condyle and medial tibial plateau. In addition, the axial imaging at the level of the suprapatellar pouch demonstrated reduction in the size of the knee effusion. The patient displayed a marked functional improvement of 43% in the Lysholm Knee score at 4 weeks after the last injection, reporting a score of 65. Similarly, a robust recovery of pain was demonstrated, with the patient reporting a pain score of 0 on the NRS. During the PPS treatment and follow-up period, the patient did not present with any drug-related or nondrug-related adverse responses.

## Discussion

The MRI findings performed using a 3-Tesla proton density fat saturated SPACE acquisition with TR 1200 ms TE 28 ms demonstrated that a course of intramuscular PPS produced complete resolution of the BML at the medial femoral condyle and medial tibial plateau. In addition, the axial imaging at the level of the suprapatellar pouch demonstrated reduction in the size of the knee effusion. The effects of PPS may be attributed to the multiple pharmaceutical actions related to its anti-inflammatory effects on IL-1 beta and TNF-alpha [20]. In addition, its inhibition of extracellular matrix degrading enzymes such as ADAMTS and MMPs [19] and improvement of microcirculation may have assisted in the reduction of cellular infiltration and minimization of the intra-osseous pressure in the bone marrow with alleviation of pain [21]. Extensive supportive data on the anti-inflammatory effects of PPS have been reported in animal and veterinary studies showing beneficial effects on the cartilage fibrillation and synovial fluid concentrations in dogs and osteoarthritic horses [17, 18]. Furthermore, a low incidence of side effects was observed in the treatment of dogs with osteoarthritis and significant reduction in cartilage degradation [25]. PPS efficacy was also shown in rat models of arthritis with the rodents showing reduced joint swelling and inflammation [26]. In support of improved microcirculation, PPS administration in rabbit and canine models exhibited increased blood flow through subchondral capillaries of osteoarthritis joints and bone cell nutrition [26].

The patient did not report any adverse effects due to PPS administration which supports the safety profile of this agent as reported in previous clinical trials [17, 22] conducted in osteoarthritic subjects. However, the new finding in this case report not previously reported were the effects of PPS on the MRI diagnostic features of subchondral BML and joint effusions. Moreover, the observed improvement in knee function in this patient may be ascribed to the chondro-protective effects of PPS [21, 22].

Current treatment options for BML associated osteoarthritis are diverse with limited reports of improved clinical outcome. The standard treatment of BML in osteoarthritic patients consists of analgesic or anti-inflammatory medications combined with reduced weight-bearing and physical therapy until symptoms reduce or resolve [27]. However, non-steroidal anti-inflammatory drugs (NSAIDs) and corticosteroids have been found to have negative effects on bone healing and the metabolism of cartilage [28–31]. As reviewed by O’Mahony [30] the risk of fracture, both traumatic and spontaneous, is increased in those subjects that take continuous corticosteroid therapy. Therefore, PPS may be a suitable pharmaceutical option to NSAIDs and corticosteroids with potential disease modifying activity. Treatment by PPS in this report demonstrated complete reduction in BML, with no post-treatment adverse events reported on follow-up with the patient.

It should be noted that different MRI sequences were used pre- and post-treatment. This occurred as the patient’s initial scan (a 2D proton density study) was an extensive diagnostic scan looking at all structures, whereas the follow-up scan (a 3D proton density SPACE study) was a more limited study specifically utilized for assessing BMLs in an efficient manner. Although there is a paucity in the literature comparing sensitivities of the two sequences in relation to BMLs and effusions, the SPACE sequence enables acquisition of high resolution 3D datasets with contrasts similar to those obtained from 2D proton density imaging [32]. Given this, it is highly unlikely the use of slightly different MRI sequences in the pre- and post-treatment MRI studies would have biased the result.

## Conclusion

Results from this case report implicate the potential effectiveness of PPS as a treatment option for osteoarthritic patients with BML and joint effusion. Given such findings, we recommend that future research seek to further investigate the efficacy of PPS in the treatment of BML associated pain and dysfunction in the osteoarthritic population via randomized controlled trial, or equivalent rigorous methodological techniques.

## Abbreviations

BML: Bone Marrow Edema Lesion
MRI: Magnetic resonance imaging
MSAIDs: Non-steroidal anti-inflammatory drugs
NRS: Numerical Rating Scale
PPS: Pentosan Polysulphate Sodium
TKA: Total Knee Arthroplasty
